# Duct Excision is Still Necessary to Rule out Breast Cancer in Patients Presenting with Spontaneous Bloodstained Nipple Discharge

**DOI:** 10.4061/2011/495315

**Published:** 2011-09-06

**Authors:** R. E. Foulkes, G. Heard, T. Boyce, R. Skyrme, P. A. Holland, C. A. Gateley

**Affiliations:** Royal Gwent Hospital, Newport, South Wales, NP20 2UB, UK

## Abstract

*Introduction*. Spontaneous nipple discharge is the third most common reason for presentation to a symptomatic breast clinic. Benign and malignant causes of spontaneous nipple discharge continue to be difficult to distinguish. We analyse our experience of duct excisions for spontaneous nipple discharge to try to identify features that raise suspicion of breast cancer and to identify features indicative of benign disease that would be suitable for nonoperative management. *Methods*. Details of one hundred and ninety-four patients who underwent duct excision for spontaneous nipple discharge between 1995 and 2005 were analysed. *Results*. Malignant disease was identified in 11 (5.7%) patients, 4 invasive and 7 insitu, which was 10.2% of those presenting with bloodstained discharge. All patients with malignant disease had bloodstained discharge. Discharge due to malignant disease was more likely to be bloodstained than that due to benign causes (Fisher's exact test, 2-tailed *P* value = 0.00134). *Conclusion*. Our findings do not support a policy of conservative management of spontaneous bloodstained nipple discharge. Cases of demonstrable spontaneous bloodstained nipple discharge should undergo duct excision to prevent malignant lesions being missed.

## 1. Introduction

Nipple discharge is the third most common symptom presenting to breast clinics, following lumps and pain [1], accounting for 3 to 10% of referrals [2]. Nipple discharge causes considerable anxiety, but it is a presenting symptom in only 5 to 12% of cases of breast cancer [2]. Suspicious discharge is described as being unilateral, single duct, spontaneous, and persistent [3]. Discharge that is clear, serous, sero-sanguinous, or bloodstained is more likely to be due to breast cancer [4].

Approximately, 55% of patients presenting with nipple discharge have an associated mass, 19% of which are malignant [3]. These patients should be investigated by the triple assessment. In some patients who do not have a palpable breast lesion, mammography identifies an abnormality which requires further investigation. The remainder will have neither a palpable nor a radiological abnormality. Where the nature of the discharge is suspicious, duct excision is required to exclude breast cancer. 

Although not routine practice in the United Kingdom, a number of techniques have been used to determine the cause of nipple discharge, beyond the triple assessment. Nipple discharge cytology has a low sensitivity for the detection of breast cancer [4, 5] and is unlikely to alter the management of patients with nipple discharge [4, 6]. Fluorescent insitu hybridization analysis of the discharge has not yet entered clinical practice however, a small pilot study has shown that it has a 100% specificity in making a definitive diagnosis of malignancy in patients with indeterminate cytologic results, suggesting that it could be a good adjunct to cytology [7].

Ductography has a high-positive predictive factor in the diagnosis of intraductal lesions, papilloma, and carcinoma; however, it has a low sensitivity and is painful [8]. Breast ductoscopy is an evolving technology, which is a promising tool as it can allow identification of the site of any lesion in younger women, allowing excision of the benign lesions while retaining the ability to lactate. However, further studies are required to define its role more clearly, as there are still limitations in clinical practice [8–15]. Magnetic resonance imaging (MRI) may play an adjunctive role, aiding in the differentiation of benign ductal abnormalities from malignant ones but remains under investigation and is not the method of choice presently in evaluating nipple discharge in the UK [16–19]. 

Only duct excision provides a definitive histological diagnosis and remains the gold standard. However, a significant number of patients with benign conditions undergo surgery, which is a concern particularly in women of child bearing age due to the implications associated with breastfeeding. The aim of this study was to analyse our experience of duct excision for nipple discharge, in an attempt to identify features that raise the suspicion of breast cancer, and to identify features indicative of benign disease where duct excision can be avoided.

## 2. Methods

All patients who underwent microdochectomy or total duct excision for spontaneous nipple discharge between 1995 and 2005 were analysed. Patients were managed by two consultant breast surgeons, who performed or supervised all surgical procedures. Data that was collected prospectively on the British Association of Surgical Oncology Database was retrieved and analysed.

During this period, 1964 patients presented with spontaneous nipple discharge, either alone or in combination with other symptoms. Triple assessment diagnosed breast cancer in 62 patients and benign causes in 1708. In the absence of a clinical or radiological abnormality to allow a definite benign or malignant diagnosis, duct excision was performed if the nature of the discharge caused concern.

**Figure 1:**
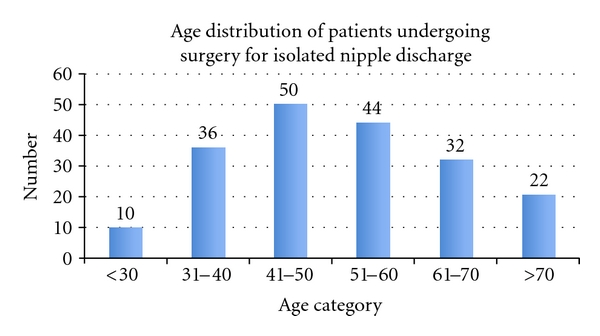
Age distribution of patients undergoing surgery for spontaneous isolated nipple discharge.

One hundred and ninety-four patients, including 1 man, underwent duct excision for spontaneous nipple discharge alone, median age 51, range 17–88 years (Figure 1). Two women had a previous history of breast carcinoma; both presented with discharge on the contralateral side to a previous mastectomy. Fifty-eight patients had a past history of benign breast disease.

## 3. Results

One hundred and ninety-four duct excisions were performed for spontaneous nipple discharge alone, 135 total duct excision, and 59 microdochectomies. Breast cancer was identified in 11 (5.7%) patients: 4 invasive and 7 insitu (Table 1). Duct ectasia and duct papilloma were the most common benign diagnoses.

The median age of patients found to have bloodstained discharge diagnosed to be breast cancer was 68 with a range of 32–88. This was higher than that for patients who were found to have benign disease, median age 50 with a range 17–84 years for patients with benign disease. 

All patients diagnosed with breast cancer, following duct excision (Table 2), subsequently underwent mastectomy with either axillary sampling or clearance. One patient was initially treated by central wide local excision, but disease-free margins could not be obtained. All 4 cases of invasive carcinoma were grade 2 or 3 invasive ductal with associated ductal carcinoma insitu. Two cases of DCIS were high grade, 4 intermediate grade, and 1 low grade. Nodal involvement was not identified in any cases. There were no major discrepancies between the histological diagnoses from the duct excision and the subsequent therapeutic surgery, in 1 case, the grade of DCIS was increased from low to intermediate. None of these patients developed recurrent disease during the 24–130 months followup.

Ten patients with breast cancer had unilateral single-duct discharge, 1 had unilateral multiple duct discharge and was found to have extensive intermediate grade DCIS. All 11 patients had some form of bloodstained discharge: 9 frankly bloodstained discharge, 1 altered blood, and 1 serous discharge that was positive for blood on dipstick testing. Discharge due to malignant disease was significantly more likely to be bloodstained than that due to benign causes (Fisher's exact test, 2-tailed *P* value = 0.001).

One hundred and eight patients (56%) who underwent duct excision had bloodstained discharge. The median age of patients with bloodstained discharge was higher than those with nonbloodstained discharge: 55, range 24–88 years, versus 47, range 17–74 years, but did not reach statistical significance (Mann-Whitney test, *P* = 0.295).

Eleven of the 108 (10.2%) patients who had some form of bloodstained nipple discharge were found to have invasive or insitu breast cancer following duct excision (Table 3). No cases of coloured discharge were associated with cancer.

Three patients were found to have atypical duct hyperplasia (ADH), of which 2 had frank bloodstained discharge and 1 serous discharge with blood on dipstick testing.

Eight women with benign pathology at duct excision have subsequently developed invasive breast cancer, 4 in the ipsilateral and 4 in the contralateral breast, including 2 of the 3 with ADH (Table 4). Based on the national registration rate for breast cancer, in the 50–54 age group, (the median age of patients in this study), in 2001 (the midpoint of this study), 3.2 breast cancers would be expected to develop during the median follow-up period of 6 years [20].

A further 24 women with benign pathology at duct excision reattended the Breast Clinic. Eighteen had symptoms on the same side as the duct excision, 4 contralateral, and 2 bilateral (1 multiple papillomatosis and 1 requesting bilateral reduction mammoplasty). The symptoms and signs at representation were similar to the original pathologies in 13 (54.2%), which was most commonly sepsis, previous surgery having demonstrated subclinical periductal mastitis.

## 4. Discussion

The management of patients with spontaneous bloodstained nipple discharge in the absence of other detectable abnormalities remains controversial. Reports have not shown an increased risk of breast cancer in patients with nipple discharge, with no other abnormality on triple assessment [21]. Several policies have been proposed, including conservative management [22], or surgery for patients with suspicious or bothersome discharge [4, 21]. 

Our findings do not support a policy of conservative management of spontaneous bloodstained nipple discharge, as 10.2% of patients were diagnosed to have breast cancer in the absence of other clinical or radiological abnormality. The median age of patients with bloodstained discharge due to breast cancer was higher than that of the patients with benign disease (68 versus 55 years) but with a large overlap of ages. It has been suggested that a conservative policy could be adopted for women under the age of 40 years [2]. However, in our series this would have led us to missing a case of widespread intermediate grade DCIS that necessitated mastectomy. From our data it would appear appropriate to advocate conservative management for women under 30, but this would only have avoided 9 of 194 operations. 

Locker et al. [23] advocated conservative management of patients with all types of nipple discharge, suggesting reinvestigation 1 year after presentation. They claimed that any breast cancer not identified at presentation would still be at a very early stage when a clinical or radiological abnormality became apparent, which would not adversely affect the outcome. If this had been applied to our series, four women with grade 2 invasive breast carcinoma would have had a delayed diagnosis. It is not possible to predict when their cancers would have become clinically or radiologically detectable or what effect that this would have had on prognosis, but a delay in diagnosing breast cancer of more than three months is considered to have prognostic significance [24]. Reviews have reported that DCIS progresses to invasive cancer in 14–53% of cases over a period of at least 10 years [25], that DCIS presenting with nipple discharge tends to be extensive and has a high rate of local recurrence if treated with breast conservation [26], and that higher grades of DCIS are more likely to recur and to progress to high-grade invasive disease [27]. Only 1 patient had low-grade DCIS which would not support a policy of conservative management.

Atypical ductal hyperplasia is associated with a risk of developing breast carcinoma of around 10% within 10 years [2]. Two of the 3 women were found to have ADH following duct excision developed breast cancer within 4 years, 1 in the contralateral breast. This raises the question of whether symptomatic ADH may impart a higher risk of developing breast cancer than incidentally identified ADH. Little data is available, but ADH associated with DCIS has been reported to increase the risk of contralateral breast cancer above that of DCIS alone, and clear surgical margins at duct excision for ADH did not affect the risk of developing breast cancer [28].

A further 6 women, 4 of who had presented with spontaneous bloodstained or serous discharge diagnosed with benign diagnoses following duct excision, have gone on to develop breast cancer, 3 in the contralateral breast. This would suggest that presenting with bloodstained or serous discharge may also be a marker of an increased risk of subsequently developing breast cancer.

Dipstick testing of nipple discharge, for occult blood, to identify those who require duct excision has been described [29, 30]. Dipsticks are very sensitive, but are of low specificity. We only use them when a patient clearly gives a history of bloodstained discharge that is not confirmed on clinical examination, and then only accept +++ reading as a positive test. In our series, 1 woman with breast cancer did not have frank bloodstained discharge on clinical examination. She gave a clear history of previously having blood in the discharge, and the serous discharge identified on examination tested positive for blood on dipstick testing.

The management of spontaneous bloodstained nipple discharge remains open to optimisation. We believe that all cases of demonstratable spontaneous bloodstained discharge in patients over the age of 30 years should undergo diagnostic duct excision. However, duct excision should be avoided in the absence of blood staining in order to prevent unnecessary surgery and possible complications, as no patients were identified to have breast cancer.

## Figures and Tables

**Table 1:** Histology of duct excision specimens.

Histology	Number	% of total (195)	Median age (range)
*Malignant *	11	6%	68 (32–88)
IDC	4	2%	72 (68–74)
DCIS	7	4%	59 (32–88)
*Benign*	183	94%	50 (17–84)
Duct ectasia	76	39%	50 (22–84)
Papilloma	65	34%	56 (26–84)
Periductal mastitis	21	11%	39 (17–59)
Fibrocystic disease	12	6%	48 (35–65)
ADH	3	1%	61 (35–76)
Normal	6	3%	43 (24–67)

IDC: invasive ductal carcinoma.

DCIS: ductal carcinoma insitu.

ADH: atypical ductal hyperplasia.

**Table 2:** Breast cancers identified following surgery for spontaneous isolated nipple discharge.

Age	Discharge type	Single duct?	Initial surgery	Initial histology	Final histology	Treatment
32	Fresh blood	Yes	Micro	DCIS	Intermediate grade DCIS	Mx + ANS + recon
43	Fresh blood	No	TDE	DCIS	Extensive intermediate grade DCIS	Mx + ANS + recon
51	Fresh blood/serous	Yes	TDE	DCIS	High grade DCIS	Mx + ANS
58	Fresh blood	Yes	TDE	IDC	3 mm grade 3 IDC + extensive DCIS	Mx + ANC + recon
60	Fresh blood	Yes	Micro	DCIS	High grade DCIS	Mx + ANS
68	Fresh blood	Yes	TDE	IDC	3 mm grade 2 IDC + DCIS	Mx + ANC
69	Fresh blood	Yes	Micro	DCIS	Multifocal intermediate grade DCIS	Mx + ANS
72	History of fresh blood; serous discharge identified, dipstick +++ for blood	Yes	TDE	IDC	10 mm grade 2 IDC + DCIS	Mx+ ANC
74	Altered blood	Yes	TDE	IDC	10 mm grade 2 IDC + widespread DCIS	Mx and ANC
78	Fresh blood	Yes	TDE	DCIS	Multifocal intermediate grade DCIS	Simple Mx
88	Fresh blood	Yes	TDE	DCIS	Low grade DCIS	Simple Mx

Initial operation: Micro: microdochectomy; TDE: total duct excision.

Histology: DCIS: ductal carcinoma insitu; IDC: invasive ductal carcinoma.

Treatment: Mx: mastectomy; ANS: axillary node sampling; ANC: axillary node clearance; Recon: reconstruction.

**Table 3:** Histology compared to type of discharge.

Histology	Number	Number with frank bloodstained discharge (% of diagnostic group)
All	194	108 (56%)
*Malignant*	11	11 (100%)
IDC	4	4
DCIS	7	7
*Benign*	183	97 (53%)
Duct ectasia	76	43
Papilloma	65	36
Periductal mastitis	21	8
Fibrocystic disease	12	5
ADH	3	2
Normal	6	4

**Table 4:** Patients with benign diagnoses at duct excision, who subsequently represented with breast cancer.

Age at original presentation	Initial presentation	Initial diagnosis	Time to re-presentation	Side	Subsequent presentation	Further histology	Treatment
63	Bloodstained single-duct nipple discharge	ADH	16 months	Contralateral	Serous nipple discharge dipstick +++ for blood	2 mm grade 2 node negative IDC +DCIS + duct papilloma	Mx + ANC
77	Bloodstained single-duct nipple discharge	ADH	48 months	Ipsilateral	Asymmetric density seen on screening mammogram follow up	16 mm grade 2 node negative IDC	Mx + ANC
49	Serous single-duct nipple discharge	Duct papilloma, duct ectasia and fibrocystic disease	84 months	Contralateral	Breast pain and nodularity	23 mm grade 2 node negative IDC + DCIS	Mx + ANC + reconstruction. Recurrence in reconstructio 15 months later treated with WLE + DXT (15 mm grade 3 IDC)
52	Bloodstained single-duct nipple discharge	Fibrocystic disease	94 months	Ipsilateral	Lump in axilla	Metastatic adenocarcinoma from presumed occult breast primary	ANC
59	Bloodstained single-duct nipple discharge	Duct ectasia	54 months	Ipsilateral	Lump	22 mm grade 2 node negative IDC	WLE + ANC + DXT
62	Brown single-duct nipple discharge	Duct papilloma	56 months	Ipsilateral	Bloodstained nipple discharge	Two adjacent <5 mm grade 2 node negative IDC	Mx + ANC
67	Serous single-duct nipple discharge	Duct papilloma	26 months	Contralateral	Bloodstained nipple discharge and nodularity	13 mm grade 2 node negative IDC, DCIS + multiple duct papillomas	WLE + ANS + DXT
76	Clear single-duct nipple discharge	Duct papilloma + duct ectasia	6 weeks	Contralateral	New lump	14 mm grade 2 node negative IDC	Mx + ANC

Histology: DCIS: ductal carcinoma in situ; IDC: Invasive ductal carcinoma.

Treatment: Mx: mastectomy; ANS: axillary node sampling; ANC: axillary node clearance; WLE: wide local excision; DXT: radiotherapy.

## References

[1] Beechey-Newman N, Kulkarni D, Kothari A (2005). Breast duct microendoscopy in nipple discharge: microbrush improves cytology. *Surgical Endoscopy and Other Interventional Techniques*.

[2] Mansel RE, Webster DJT, Sweetland HM (2009). *Benign Disorders and Diseases of the Breast*.

[3] Florio MG, Manganaro T, Polllcino A, Scarfo P, Micali B (1999). Surgical approach to nipple discharge: a ten-year experience. *Journal of Surgical Oncology*.

[4] King TA, Carter KM, Bolton JS (2000). A simple approach to nipple discharge. *The American Surgeon*.

[5] Cabioglu N, Hunt KK, Singletary SE (2003). Surgical decision making and factors determining a diagnosis of breast carcinoma in women presenting with nipple discharge. *Journal of the American College of Surgeons*.

[6] Wahner-Roedler DL, Reynolds C, Morton MJ (2003). Spontaneous unilateral nipple discharge: when screening tests are negative—a case report and review of current diagnostic management of a pathologic nipple discharge. *Breast Journal*.

[7] Yamamoto D, Senzaki H, Nakagawa H, Okugawa H, Gondo H, Tanaka K (2003). Detection of chromosomal aneusomy by fluorescence in situ hybridization for patients with nipple discharge. *Cancer*.

[8] Goksel HA, Yagmurdur MC, Demirhan B (2005). Management strategies for patients with nipple discharge. *Langenbeck’s Archives of Surgery*.

[9] Louie LD, Crowe JP, Dawson AE (2006). Identification of breast cancer in patients with pathologic nipple discharge: does ductoscopy predict malignancy?. *The American Journal of Surgery*.

[10] Escobar PF, Crowe JP, Matsunaga T, Mokbel K (2006). The clinical applications of mammary ductoscopy. *The American Journal of Surgery*.

[11] Dooley WC (2002). Routine operative breast endoscopy for bloody nipple discharge. *Annals of Surgical Oncology*.

[12] Denewer A, El-Etribi K, Nada N, El-Metwally M (2008). The role and limitations of mammary ductoscope in management of pathologic nipple discharge. *Breast Journal*.

[13] Simpson JS, Connolly EM, Leong WL (2009). Mammary ductoscopy in the evaluation and treatment of pathologic nipple discharge: a Canadian experience. *Canadian Journal of Surgery*.

[14] Kamali S, Bender O, Aydin MT, Yuney E, Kamali G (2010). Ductoscopy in the evaluation and management of nipple discharge. *Annals of Surgical Oncology*.

[15] Dooley WC (2009). Breast ductoscopy and the evolution of the intraductal approach to breast cancer. *Breast*.

[16] Hirose M, Otsuki N, Hayano D (2006). Multi-volume fusion imaging of MR ductography and MR mammography for patients with nipple discharge. *Magnetic Resonance in Medical Sciences*.

[17] Tokuda Y, Kuriyama K, Nakamoto A (2009). Evaluation of suspicious nipple discharge by magnetic resonance mammography based on breast imaging reporting and data system magnetic resonance imaging descriptors. *Journal of Computer Assisted Tomography*.

[18] Lorenzon M, Zuiani C, Linda A, Londero V, Girometti R, Bazzocchi M (2010). Magnetic resonance imaging in patients with nipple discharge: should we recommend it?. *European Radiology*.

[19] Ballesio L, Maggi C, Savelli S (2008). Role of breast magnetic resonance imaging (MRI) in patients with unilateral nipple discharge: preliminary study. *Radiologia Medica*.

[20] Office for National Statistics (2004). *Cancer Statistics: Registrations of Cancer Diagnosed in 2001, England*.

[21] Vargas HI, Romero L, Chlebowski RT (2002). Management of bloody nipple discharge. *Current Treatment Options in Oncology*.

[22] Richards T, Hunt A, Courtney S, Umeh H (2007). Nipple discharge: a sign of breast cancer?. *Annals of the Royal College of Surgeons of England*.

[23] Locker AP, Galea MH, Ellis IO, Holliday HW, Elston CW, Blamey RW (1988). Microdochectomy for single-duct discharge from the nipple. *British Journal of Surgery*.

[24] Richards MA, Westcombe AM, Love SB, Littlejohns P, Ramirez AJ (1999). Influence of delay on survival in patients with breast cancer: a systematic review. *The Lancet*.

[25] Erbas B, Provenzano E, Armes J, Gertig D (2006). The natural history of ductal carcinoma in situ of the breast: a review. *Breast Cancer Research and Treatment*.

[26] Winchester DP, Jeske JM, Goldschmidt RA (2000). The diagnosis and management of ductal carcinoma in-situ of the breast. *CA Cancer Journal for Clinicians*.

[27] Ottesen GL, Graversen HP, Blichert-Toft M, Christensen IJ, Andersen JA (2000). Carcinoma in situ of the female breast: 10 Year follow-up results of a prospective nationwide study. *Breast Cancer Research and Treatment*.

[28] Greene T, Tartter PI, Smith SR, Estabrook A (2006). The signficance of surgical margins for patients with atypical ductal hyperplasia. *The American Journal of Surgery*.

[29] Dixon JM, Mansel RE (1994). ABC of breast diseases: symptoms assessment and guidelines for referral. *British Medical Journal*.

[30] Chaudary MA, Millis RR, Davies GC, Hayward JL (1982). Nipple discharge: the diagnostic value of testing for occult blood. *Annals of Surgery*.

